# Editorial

**Published:** 1864-02

**Authors:** 


### Editorial.

Illinois State Medical Society.—The next regular Annual Meeting of the Illinois State Medical Society will be held in Chicago, commencing on the first Tuesday of May next. We anticipate a full attendance and profitable meeting on that occasion.

Chicago Medical College Commencement.—The Fifth Annual Commencement of the Chicago Medical College—Medical Department of Lind University—will be held on the evening of the first Tuesday in March next.

Books and Pamphlets.—Several of both have been received, and will be duly noticed in our next number.

Sarracenia Purpurea in Small-Pox.—In another part of this number we publish the result of the experience of Dr. N. C. Levings, in the use of this article. We have also used it in several cases. In the majority of them we could not perceive that it had any effect whatever. In one case, of a young man who supposed he had been well protected by vaccination, but on whom the variolous eruption made its appearance so thick as to indicate a severe form of the disease, we gave the infusion of the Sarracenia freely, and the entire eruption proved abortive, drying up completely about the fifth day without suppurating. No doubt such occurrences have been noticed by others, even where the Sarracenia has not been used, and hence they must be regarded as purely accidental.

Commencement in Rush Medical College.—The commencement exercises of the twenty-first annual course of lectures in Rush Medical College were held on Wednesday evening, the 27th ult., on which occasion seventy-nine gentlemen received the Degree of Doctor of Medicine. Prof. Brainard, President of the Faculty, conferred the degrees, presenting each graduate with his diploma.—*Chicago Medical Journal.*

STATISTICS OF MORTALITY IN CHICAGO.—As some of our readers may have seen the pretended tables of mortality published in the daily papers of this city once a month, we insert the following communication, sent by the Chicago Medical Society to the Mayor and Council, for the purpose of relieving the profession of the city from any responsibility for the imperfections and absurdities of these tables. The communication was received in the Council and referred to the Judiciary Committee, with a fair prospect of having better regulations for the future:—

To the Mayor and Council of Chicago:—

The Chicago Medical Society would respectfully call your attention to the defective method of obtaining and recording the statistics of mortality in this city, which has been adopted for several years past.

It is justly considered one of the most important duties of every municipal government to ascertain and remove, as far as practicable, all causes of disease and death in the community under its jurisdiction.

In the work of ferreting out the causes of disease in any given city or community, a correct record of mortality, setting forth the age, sex, nativity, and place of residence, with the immediate cause of death in each case, is indispensably necessary. Such a record in this city would enable the medical faculty and the Health Department to determine at all times, not only the total number of deaths, but the precise streets and wards in which they occurred, the class of people suffering most, and the nature of the diseases giving rise to the mortality. These facts being ascertained, a comparison of the sanitary condition of the streets and wards furnishing the highest ratio of mortality with that of the more healthy districts, would quickly show the causes acting injuriously upon the health of the people, and how far they were capable of removal.

For the purpose of furnishing such a record of mortality as we have here suggested, the framers of the present city charter and ordinances made the following provisions, viz.:—

"SEC. 12. It shall be the duty of the City Sexton to keep a record of all interments which may be made in the city: stating the name, age, and disease, or other cause, and date of death, the *Division* in which the deceased resided, and when practicable, the place of birth. A transcript of said record shall be filed by the Sexton in the City Clerk's office for record, at least once a month; and when required, he shall cause the same to be published.

"SEC. 13. For the more effectual execution of the foregoing section, it shall be the duty of the attending physician, on the demise of his patient, to grant a certificate to the sexton in attendance, on application, setting forth the particulars above specified. Said certificate shall be delivered previous to interment, to the City Sexton by the sexton in attendance. And in all cases where no physician shall have been in attendance, or where the attending physician shall neglect or refuse to grant such certificate, or cannot be found, it shall be the duty of the sexton to ascertain and report in writing to the City Sexton, previous to interment, the several particulars aforesaid, and the name of any physician who shall refuse or neglect to grant said certificate. If any sexton or physician shall fail to comply with any provision hereof, he shall be subject to a penalty of five dollars for every offence. Every person shall be deemed a sexton who shall officiate as such at any funeral. The City Sexton is hereby authorized to procure suitable blanks for the use of physicians and sextons, and furnish the same gratis."

With one exception, these provisions are sufficient, if correctly executed according to their plain intent and meaning, for all the purposes desired. The exception to which we allude, has reference to the locality of each death. The law as it now stands requires only the specification of the "Division" of the city in which it occurred. With the present extended limits of the city this is too vague to be of any practical utility. Every certificate of death should state the street and number, or if no number exists, the street with the name of the nearest street crossing it. Such an amendment would enable the Health Officer at all times to determine the precise district or neighborhood in which any unusual mortality was occurring.

Knowing fully the paramount importance of this subject, to the welfare and prosperity of the city, we earnestly ask your attention to the amendment here proposed, and then to a rigid and faithful enforcement of all the provisions of the law. The record of mortality as it has been kept in this city for five or six years past, is entirely worthless, except in relation to the gross mortality of the city. We are aware that for some time past a tabulated monthly statement has been published, pretending to set forth not only the gross mortality for the month, but also the causes of death. The undertakers and sextons, instead of procuring reliable certificates, in accordance with the law, from the attending physicians, simply gather from the family or neighbors some pretended cause of death, insert it in the certificate themselves, and return it for registry. So true is this that not a single member of this Society has been called on for a certificate of death twice a year for the last five years. Of course certificates made up in the manner here indicated are mere inventions; and the tabular statements founded on them and published, are so grossly absurd as to be a disgrace to the Health Department of the city. That we do not exaggerate will be apparent by a reference to any of the published tables. Take, for instance, the most recent statement, which professes to include the causes of death for the year 1863. There we have six deaths ascribed to "abscess;" but in what part of the body is not stated. Thirty-four are ascribed to "accident;" yet further along in the table, twenty-nine more are recorded as having been "killed," and fifty-five "drowned." Forty-four are said to have died of "decline;" but, whether this means that they died because they "*declined*" to live any longer, or because they had consumption, it is not possible to determine. Seven died of "fever," and twenty-one of "fits," but what kind of *fever* or *fits* is not stated. Seven died from "lung disease," five because they were "*nervous*," one hundred and eighty-six from "teething," &c., &c. It is needless to repeat that such tables are not only valueless, but they are subjects of jest and ridicule, both at home and abroad. The faults of these tables are not to be ascribed to the Health Officer, who nominally reports them, but to the sextons who *manufacture* certificates of death, instead of procuring them from physicians, as the law requires. To correct the evils and negligence so manifest in this important department of our city affairs, we earnestly ask your attention to the following propositions:—

1st. Amend section 12 of revised ordinances, relating to the Health Department, by striking out the word "division," and inseting in its place the words "street, number, and ward."

2d. Adopt such measures as will insure the faithful execution of all the provisions of the law in relation to the procurement of proper certificates, their prompt return to the proper officer, and their regular tabulation and publication.

N. S. Davis, *Chairman of Committee.*

---

The Chair of Chemistry at Berlin, also that at Bonn, have been offered to Dr. Hoffman, of London. The University of Bonn propose to place £20,000 at his disposal for the establishment of a laboratory.

### STATE BOARD OF EXAMINERS FOR THE DEGREE OF DOCTOR IN MEDICINE.

We are gratified to see that the initiative in this measure has been taken by the Faculty of the Buffalo Medical College, and most earnestly hope it may cause the adoption, in tangible form, of the measure, which if carried out will do more for the elevation of the profession than any other reform which could be suggested.

At the Annual Meeting of the New York State Medical Society, now being held in Albany, the following was presented, and on motion adopted:—

UNIVERSITY OF BUFFALO, MEDICAL DEPARTMENT.

On motion of Prof. Chas. A. Lee, seconded by Prof. James P. White, it was

*Resolved*, That the New York State Medical Society be requested to appoint a committee to consider the expediency of, and to report a plan for, the appointment of a State Board of Examiners for the degree of Doctor of Medicine, and to report at the next meeting of the Society.

*Resolved*, That the same committee be instructed to bring the subject before the next meeting of the American Medical Association, and that the delegates of this Society be instructed to urge the general adoption of the same plan in other States of the Union. Carried unanimously.

THOS. F. ROCHESTER, *Chairman.*

SANFORD EASTMAN, *Dean of the Faculty.*

*Buffalo, Feb. 2d*, 1863.

This is certainly an effort in the right direction, and is exceedingly creditable to the institution suggesting it. When the graduation of students in medicine is wholly separated from the duty of teaching, and an impartial Board of Examiners shall decide who shall, and who shall not receive the degree of Doctor in Medicine, very much will be accomplished for the elevation and advancement of the profession. It is striking at the very root of a great evil, and will meet with opposition; indeed, we have no doubt it will be overwhelmed in the almost unanimous opposition which it will meet from the various institutions now empowered to grant diplomas. It is a measure that Medical Colleges, as such, cannot at present afford to favor, though the Professors are more fully sensible of its importance than any other individuals. There is no doubt that an impartial Board of Examiners would reject, as unprepared for the duties of the medical profession, from one-quarter to one-third of the young men who, under the present system of graduation, are yearly admitted to the practice of medicine. This we believe to be true, and to be more or less applicable to all places in this country where young men are taught the primary branches of medical knowledge, and graduate, after "three years' study, and two full courses of lectures, the last at this institution." The State Examiners should receive compensation only from the State; should be disconnected from all schools of instruction; should, in a word, be wholly impartial.

It has been claimed that the same was accomplished by the appointment of Curators, who are invited to *attend* the examination of the students and vote upon their qualifications; and this does *appear* quite satisfactory, while in reality it accomplishes nothing. Whoever notices the manner of these appointments, the terms of this service, and the opportunity afforded to determine the respective merits of individuals, will readily discover that in this way the feeblest protection is afforded the open doors of our profession, which should be guarded by ever faithful janitors.

We understand, also, that this plan of appointing State Examiners has been considerably perfected by the Institution suggesting it, and that the details will be urged upon the consideration of the State Society at its present meeting. It appears that the Buffalo Medical College are in earnest in advocating this measure. The competition in medical schools and the desire to obtain large classes, has had prejudicial influence upon the standard of medical education, and so great has this evil become, that it is quite time for commencing a reform. Young men have been encouraged to pursue the study of medicine without preliminary preparation, and graduated without respectable professional attainment, thus lowering the professional standard, and making the degree of Doctor in Medicine a disgrace, rather than an honor. If this reform, now suggested and urged upon the profession by the Medical College in Buffalo, is favored by the other colleges in the State, we shall soon be redeemed from the power and influence of a system which has disgraced the profession, lowered its standard of attainment, reflected obloquy and contempt upon its degree, and come well nigh reducing medicine, as learned and practiced, "to the level of a trade."

It may be thought that we draw this matter in rather high colors, but the revelations of modern investigation leave no doubt that a poor doctor is vastly worse than none at all. I say revelations of modern investigation, for it was formerly believed that any one who could give some medicine was useful in the absence of a physician; but it has been reserved for the physicians of our day to discover, and for the people of our times, in any measure to perceive, that medicine, *per se*, is poison, and useful only when applied under the direction of an honest, educated, intelligent physician. We sincerely hope the profession will see to it that no other than such are hereafter admitted to its ranks.—*Buffalo Medical Journal.*

We copy the above from the *Buffalo Medical and Surgical Journal*, for the purpose of giving it our cordial and earnest approval. The establishment of an efficient Board of Medical Examiners in each State, by whom all candidates should be carefully and thoroughly examined, before they receive the degree of *Doctor of Medicine*, or can be admitted into the ranks of the profession, we have earnestly advocated, on all suitable occasions, since 1840. Some of the phraseology used by the editor of the *Buffalo Journal*, would lead the reader to infer, that this was the first time the subject had been introduced into the New York State Medical Society, and that the important proposition to separate the examining and licensing boards from the Medical Colleges, had originated with the Faculty of the Buffalo Medical College. Such an inference, however, would be entirely erroneous, as any one can ascertain by referring to the transactions of that Society from 1839 to 1845. It was in reference to this very subject, that the first call was issued for organizing the American Medical Association; and that organization will not have accomplished the most important object of its existence, until it shall have prompted the establishment of an independent and efficient Board of Examiners in each State, by which all candidates must be examined and approved, as the only means of getting access to the ranks of the Medical Profession.

In 1851, we published a small volume, entitled "History of Medical Education and Institutions in the United States," in which we gave our views concerning the whole subject of medical education without reserve, and, though we have been constantly connected with medical colleges since, we cannot now better express our sentiments concerning Boards of Examiners, than by quoting from that work as follows:—

"Hence, a thorough organization of the profession should be the first object of every advocate for medical improvement; and this organization should include—first, a board of censors, appointed by each local society, to examine all candidates for admission as students, in regard to their preliminary education; and no member of such societies should admit a student into his office without a certificate from said board, certifying that he is well versed in all the branches usually taught in our academical institutions, and possesses a good moral character. And second, one board in each State for the examination of all candidates for full admission into the ranks of the profession.

"This board should consist of, at least, seven members, appointed by the State Medical Society of each State; and, if advisable, also, one additional member, appointed by each regularly incorporated medical college; and the presence of two-thirds should constitute a quorum for the transaction of business. The board should meet at such time and place as the State Society should direct, and should not only require of each candidate the ordinary oral examination in the various branches of medical science, but also, the presentation of a written thesis on some medical subject, the detailed report of one or more cases, and the examination of at least one patient, in the presence of the board. The examination, and all the requirements, should be the same, whether the candidate possesses a diploma conferred by a medical college, or not. All who are found qualified should receive from the board diplomas, certifying to such qualifications, and entitling them to be recognized as members of the profession throughout the whole country; but without such diploma, no one should become eligible to membership in any society, or be countenanced or consulted with as practitioners. All fees derived from the granting of diplomas should be paid directly into the treasury of the State Society by which the examining board was appointed; and the members of such board should be paid a reasonable compensation for the time actually spent in the performance of their duties, as examiners, by the same society,—their bills, duly certified to, being presented to a regular meeting of the society, and audited in the same manner as provided for all other bills of expenditure.

"The advantages of such a plan, when carried into practical operation, are manifold:—

"1st. It would secure the practical adoption of a fair standard of preliminary education, which is as essential to the elevation and usefulness of the profession as is a knowledge of geography to the naturalist.

"2d. It would insure a more uniform, elevated, and practical standard of requirements for admission into the ranks of the profession; because the several State societies being directly connected with each other, through the medium of the National Association, would almost necessarily give to their several boards of censors similar rules and exactions.

"3d. It would place the responsibility of fixing the qualifications, and regulating the admission of members into the profession, where it rightfully and properly belongs, viz., with the mass of the profession itself.

"4th. It would tend greatly to elevate the character of medical teaching, both public and private, by making every teacher, and every faculty of teachers, depend entirely on their *merits* for success. Mere speculating associations, or sham corporations, would no longer be able to draw respectable classes of students by the cheapness of their diplomas, the liberality with which they are distributed, or the shortness of time required for college attendance; but the student, knowing that the success of his final examination must depend entirely on the amount and readiness of his medical knowledge, his mental discipline, and his moral character—uninfluenced by the fact that he has spent more or less time and money in this or that college, or the question, whether his approval or rejection will benefit or injure this or that institution—he will be governed, in his choice of teachers and colleges, by one simple question, viz.:—Where can I gain the *greatest amount* of sound medical knowledge for a given amount of time and money? With this question as the sole issue between the teacher or the college and the pupil, we should speedily have a radical change in the nature of the competition among our medical institutions. Instead of a struggle to outdo each other in placing the diploma in such a position, or on such terms, as to be most effectual in decoying students into their own halls, their competition would necessarily consist in an effort to excel in the number of their teachers, and the length and perfection of their courses, compared with their charges—a competition tending, necessarily, to progression and improvement, instead of the reverse, which now prevails. Indeed, nothing hangs as a heavier incubus on all attempts to improve our system of medical education, than this connection of licensing and teaching. It enables the merest shadow of a college, with its thirteen or fourteen weeks' lecture term, and perhaps two of these in one year, to issue diplomas just as large, couched in just as flourishing Latin, conferring just as many privileges, and, as the student well knows, having just as much influence with the great mass of the community as the best, most thoroughly organized, and most rigid institution in the Union—hence, one of the strongest motives to real excellence in teaching is done away with, and the poorest college is given a decided advantage over the best.

"It is on this ground that I have, for several years, urged this separation, as a measure of real benefit to the colleges themselves, and as the only one which would ever enable the good institutions to reap the full benefit of their merits, in opposition to the badly managed and worthless. Indeed, the whole history of mankind, in all ages and countries, has not more clearly demonstrated the truth of any proposition than this—that every class of institutions, whether educational or industrial, not only flourish best, but are most progressive and improving in their condition, when left to depend entirely on their own merits for patronage and success.

"5th. It would insure both permanence and efficiency in the social organization. Those who study carefully the history of those medical societies and associations which have been formed, from time to time, in different States, will not fail to perceive that, with a very few exceptions, they have flourished for a few years only, and then maintained a nominal, rather than an active, state of existence. Thus, from 1810 to 1830, an active spirit of medical organization prevailed, resulting in the formation of State and County or District Societies, in a large majority of the States then existing in the Union. At first, many of these had Boards of Censors, whose fees, derived from the examination and licensing of students, not only defrayed their ordinary expenses, but, with a trifling iniatory tax, served to accumulate valuable society libraries, and the interest felt in their regular meetings continued unabated. But, as already shown in the historical part of our work, the college diploma rapidly superceded the State and county licenses, in all the States except, perhaps, Delaware and Louisiana, where a State license is still required by law.

"The societies being thus left to depend entirely on the voluntary contributions of the practitioner, who must tax himself to support the county society, spend his time, and tax himself again to pay traveling expenses, and to sustain the State organization, soon began to lose their interest, and fall into a state of inactivity. So true was this, that, though State and District Medical Societies had previously been formed in all the Eastern States—in New York, New Jersey, Delaware, Maryland, Mississippi, Alabama, Tennesse, Ohio, Indiana, and Michigan—

yet, in 1840, those in Massachusetts and New York were almost the only ones that maintained anything more than a mere nominal existence. And even in the latter State, out of its sixty counties, not more than sixteen or seventeen were represented in the meetings of the State Society. Since the successful organization of the American Medical Association, and the general interest which has been excited on the subject of medical education, a new and active spirit of social organization has been rekindled, Hence, during the last four years, most of the old societies have been re-animatod, and new ones have been formed in most of the States, where none existed before, such as Pennsylvania, South Carolina, Georgia, Illinois, Iowa, and Wisconsin. As proof of the present activity of this spirit, it is only necessary to mention, that over four hundred delegates were in attendance on each of the last two meetings of the American Medical Association, the one in Boston, the other in Cincinnati, and that many of these traveled more than a thousand miles, at their own expense, for that purpose. But does any one suppose that this spirit will continue year after year, under the influence of such personal sacrifices of time and money? It requires only an ordinary knowledge of human nature, and of the past history of medical associations, to see clearly that, without some collateral aid, some permanent resource for lightening the burthens and increasing the interest of such organizations, they will inevitably sink into a mere nominal existence, so soon as the exciting subjects which brought them into being cease to be the predominant topics of interest. But if each State organization could receive an annual income sufficient to defray its ordinary expenses, publish its transactions, and, perhaps, enable it to offer a premium for original essays, or defray the expense of original experimental investigations, it would not only insure the permanent prosperity of such organization, but it would prove one of the most powerful means of improving the whole literature and science of the profession.

"One of the primary objects I had in view when I presented the first series of resolutions in the New York State Medical Society, in 1844, advocating the separation of the licensing from the teaching power in onr colleges, and investing it in State censors, was to put into the possession of the State Societies such an income, for precisely such a purpose. That this was a leading object, will be seen by a reference to the resolutions themselves, as published in the transactions of that Society.

"It will be seen by reference to the report of the committee on medical education, contained in the Transactions of the American Medical Association for 1849, that nearly fourteen hundred were admitted into the profession during the year previous, by receiving diplomas from the several medical colleges in our country. These, at twenty dollars each, gave those colleges no less than twenty-eight thousand dollars. Now, suppose this sum was annually received by boards of examiners, one for each State, and turned directly into the treasury of the State Societies, every reader will see that it would afford an ample fund for paying the examining boards, publishing the annual transactions of the several societies, and enable each to powerfully encourage original investigations by premiums or experimental committees; and who can calculate the beneficial results that would accrue to the whole profession by thus rendering its organizations permanent and prosperous, and maintaining an active and ever-increasing spirit of scientific inquiry? It may be said that the loss of this fund would, by crippling the colleges, injure the cause of medical education as much as it would advance it, in the manner proposed; but if we remember that there are about four thousand five hundred students annually in attendance on the several colleges, and that a matriculation fee of five dollars for each, after deducting five hundred as third course students, would give no less than twenty thousand dollars, besides the entire receipts for lecture fees, we shall be satisfied that these institutions would have no cause to complain. And even if such a course should cause a school, here and there, to close its doors, it is by no means certain that either the profession or the community would suffer thereby."

Such were our sentiments years ago, and such they are still. We are glad the representatives of the Buffalo Medical College in the New State Medical Society have, again, brought the subject to the notice of the Profession. We wish the Medical Society of this, and all the other States, would give the subject a thorough consideration, and so instruct their delegates to the American Medical Association, as to effect a uniform plan of action throughout the whole country.

---

Presidency of Guy's Hospital.—At a general court of the governors, held on Wednesday, the 16th ult., the Right Hon. Sir Lawrence Peel was unanimously elected president of the institution, in the room of the late Mr. Bonamy Dobree.

### DEATH OF LEONIDAS M. LAWSON, M.D.

It is our sad duty once more to record the decease of one of our most prominent professional brothers. Dr. L. M. Lawson—late Prof. of the Theory and Practice of Medicine in the Medical College of Ohio—died at his residence in this city at one o'clock on Thursday morning, January 21st, at the early age of fifty-one.

Dr. Lawson was throughout his professional life identified with the interests of the profession of Cincinnati and medical teaching in our city, nevertheless he had occupied various positions of honor in neighboring cities at different periods of time. Very early in his career he was elected to a professorship in the medical department of Transylvania University at Lexington, Ky. Subsequently he held a professorship in Louisville for two or three winters, and for a single winter, (1859–60), he was Prof. of Clinical Medicine in the University of Louisiana at New Orleans. Still with these honorable appointments we find his heart regularly returning its best affections to this city of his early adoption. Here he has done his best work; here he has closed his labors.

In the spring of 1842, Dr. Lawson established the *Western Lancet*, and continued at its head, with various associates, until the winter of 1854–55, when his absence in Louisville made it necessary for him to withdraw from his editorial duties here. The subsequent merging of the *Medical Observer* with the *Lancet* as *Lancet and Observer*, of course renders this the regular successor of Dr. Lawson's founding in 1842. A present tribute of respect, therefore, comes from no one with more propriety, certainly with no greater sincerity and esteem for his professional industry and scholarship, and for his many social and domestic virtues, than from us.

Immediately after returning from New Orleans, Dr. Lawson brought out his work on Phthisis Pulmonalis, the labor of his life. We quote the following closing paragraph of the *critique* of the *British and Foreign Medico-Chirurgico Review* in its notice of Dr. Lawson's book, April, 1863:

"For acuteness of observation, for sober discrimination and sound judgment, and fair criticism of the writings of others, and especially of cotemporaries, and for the wide knowledge which it displays of the literature of his subject, we know few books superior to it. We bestow our praise the more readily, our author being an American, of Anglo-Saxon race, as his name implies, and one who, we trust, will, with all his right-minded countrymen, still cherish a love of the old stock from which he sprang, abhorrent of the vulgar clamor sadly now prevailing against England, as if the American States, whether united or separated, Federal or Confederate, had not, with our county, a common interest, apart from the community of blood—that of language, of literature, and of laws."

Dr. Lawson continued in the regular performance of his professional and college duties up to the time of the Christmas holidays, though it was well known that his health was feeble, and that study and close attention to duty was telling upon him. He then went to the country for a brief relaxation, but returned after a few days to take his sick couch, from which he was destined never more to return to the labors of earth.

Dr. W. H. Taylor, who conducted the *post mortem* examination, has handed us the following notes, which will be read with interest.

*Examination Thirty-Six Hours after Death.*—Body emaciated, anæmic, slight *post mortem* rigidity. Extensive adhesions of the pleura were found, which in the upper part of the thorax were very firm, in the lower lateral portions of left were indications of recent inflammation. The lungs presented extensive vesicular emphysema predominating in the right. In the apex of right lung were several tubercular cavities each about the size of a hazel nut. Throughout the entire parenchyma of both lungs were small yellow tubercles in all stages, some hard, some softening, some cretified. The surrounding lung structure was engorged, and in some portions hepatized. The pericardium was healthy. It contained rather more than the usual amount of fluid which was tinged with blood. The walls of the heart were not more than half their usual thickness, and were so soft as to be easily penetrated by the finger. The small intestines were healthy. In the head of the colon were numerous small oval and round ulcers penetrating the mucus and muscular coats. The mucus membrane surrounding the ulcers was of a dark color. Several patches of chronic engorgement were found in the mucus membrane of the rectum. The liver was so soft as to tear by its own weight when but partially raised. The spleen was twice its usual size and very soft. The kidneys were about normal size, dark colored, very flabby, and the fascia propria easily detracted. On section the junction of the cortical and medullary portions was scarcely distinguishable. A considerable quantity of thin dark fluid with oil globules flowed from the cut surface. The calices were lined by a yellow deposit of cheesy consistence about a line in thickness, and contained a milky fluid.

The following is the tribute of the profession on this occasion:

In Memoriam.—At a meeting of the Regular Medical Profession, held at the Medical College of Ohio, on Saturday, 23d inst., the following resolutions were, after appropriate remarks, unanimously adopted.

J. L. Vattier, M.D., President.

J. P. Walker, M.D., Secretary.

"*Whereas*, It has pleased God, in his good providence, to remove from our midst our professional brother, Dr. L. M. Lawson, late Professor of Theory and Practice in the Medical College of Ohio; therefore, be it

"*Resolved*, That in the death of Dr. Lawson, the profession of this city and whole country, has lost an accomplished member, and one wholly devoted to scientific pursuits.

"*Resolved, further*, That in his death the profession has lost a member whose labors in behalf of medical science have given additional luster to the American profession of medicine at home and abroad.

"*Resolved*, That in him we lose the well-bred gentleman, of amiable manners, wholly directed during his entire life, to the advancement of his profession, and the welfare of its members.

"*Resolved*, That a copy of these resolutions be sent to the family of the deceased, and published in the daily papers, and in the Cincinnati *Lancet and Observer.*"

The funeral took place from the First Presbyterian Church of this city, the discourse being delivered by the Rev. Mr. Worrall, of Covington, and the remains were followed to the cemetery by the Freemasons, of which body he was a Knight Templar, by the Profession, and the students of the Medical College of Ohio. His memory and teachings will long remain with the profession of this Great Valley. His body rests in the tomb 'till the beauty of the Resurrection morn.—*Cincinnati Lancet and Observer.*

---

French and English Army Hospitals.—Dr. H. B. Franklyn, Surgeon First Battalion 10th Foot, in a letter to the *Times*, states that, with respect to the special diseases which form the bulk of admissions to the English army hospitals, the French are more fortunate than ourselves. In 1858, the admissions to the hospitals in Paris were 24 per 1,000 men, while at Aldershot they were 411 per 1,000, and at Woolwich 512 per 1,000. Sometimes it fell in Paris to 16 per 1,000. In Marseilles, the worst French garrison, it reached 113 per 1,000.

AZULENE OF PATCHOULY.—Those who took an interest in the exhibition of 1862 know that Mr. Septimus Piesse was one of its most active Jurors in Class 4. While examining the exhibits of M. Mero, of Grasse, an apparently anomalous substance was shown to the jurors: this body was called "chrystalized patchouly." Now, chrystalized anise and crystalized otto of roses are the normal state of these bodies, but with patchouly oil it is not so, and the jurors naturally viewed crystalized patchouly with some suspicion. Portions of it were given to Mr. Piesse for chemical examination. The result proved that the crystals were a true camphor of patchouly of the atomic composition of valerole. M. Mero got his medal. The chemical examination of otto of patchouly was continued by Mr. Piesse, and the result has been the discovery of a remarkable blue body, termed by him *azulene*, from its sky-blue tint and from its sky-like optical properties. Further study has established the fact that azulene is not peculiar to patchouly, but exists in several essential oils, to the presence of which, in fact, they own their color. Blue oils, such as from the wild chamomile, contain pure azulene; green oils, such as wormwood, contain azulene and a considerable portion of yellow resin, thus disguising its presence. Sir David Brewster has pure azulene under optical examination. Several years past he made experiments in the same direction. He says: "Two blue oils, Matricaria chamomilia and Achillea millefolium, which owe their color to the presence of azulene, differ from all the bodies which I have yet examined. Between the two lines A and B of Farnnhofer's map of the spectrum, there are two groups of lines, and the two oils absorb the light in these portions more powerfully than in the portions adjacent to them. No other fluid or solid on which I have made experiment acts in a similar manner; but, what is very remarkable, the earth's atmosphere exercises a similar action when the sun's light passes through its greatest thickness at sunrise and sunset." In a paper by Mr. Piesse, read before the Chemical Society, describing azulene, the author stated that blue otto of chamomile yields one per cent., otto of wormwood three, and otto of patchouly six per cent. of the new body. The patchouly plant is already commercially cultivated at Penang, and any quality can be grown in Ceylon. Patchouly is said to enter into the composition of Indian or Chinese ink. Mr. Piesse thinks that, on account of the general presence of azulene in volatile oils (to which, in a measure, it gives their color,) it plays some special part in connexion with these odoriferous bodies, and he hopes soon to elicit some more facts relative to it.

On Topical Injections of Strychnine in Cases of Paralysis of the Facial Nerve.—M. Courty, Professor of Surgery at the Faculty of Montpelier, having succeeded in controlling severe neuralgic pains by injections of strychnine, tried them likewise in paralysis of the facial nerve, as well as loss of the power of movement in other parts. In different cases, of paralysis, and especially in chronic cases, the result was not favorable. The author succeeded, however, in a case of paraplegia; the patient, a woman aged forty-five, having been thus paralyzed for twelve months. Many remedies had been tried; but a few injections of strychnine on a level with the inferior extremity of the spinal marrow sufficed for the cure. Success was also obtained in three cases of recent facial paralysis. The first patient was a man of fifty-six; the second, a lady of twenty-five; and the third, a young lady of twenty-two. They were all in the early stage of the disease; and the strength of the solution varied from one in a hundred to one in seventy. A few drops (from eight to sixteen) were injected along the course of the facial nerve, between the stylo-mastoid foramen and the neck of the lower maxilla. The injection was repeated every second or third day. All the muscles of the face recovered the faculty of movement after from three to six injections, in about ten days or a fortnight. The author states that no relapses have taken place in these cases.

---

New American Pharmacopœia.—The January number of the *London Lancet* contains a very appreciative review of the last edition of our Pharmacopœia. The notice concludes with the following paragraph:

"Upon the whole, we consider the New United States Pharmacopœia a work highly creditable to its compilers and the profession. It bears the impress of an honest and earnest endeavor to advance the science and art of healing, to render available to all the experience and information obtainable from every quarter, and without favor or prejudice to adopt whatever may be practically useful from any source."

---

Gossip—New Mode of Preparing Beef Tea.—A medical friend had occasion not long since to order "beef tea" for a patient, and at a subsequent visit happened to inquire of the nurse if she understood the art of making beef tea correctly: Oh, yes, she replied—but for fear she might be mistaken she had consulted another lady friend learned in the duties of the sick room; and between us, said she, we succeeded beautifully. I

took a nice piece of beef—cut it in very fine pieces—put them in a bottle, corked it carefully, and then put it in a kettle of water and boiled for two hours: we then took out the bottle and fed the patient a spoonful of the water from the kettle every two hours!

---

HEALTH IN THE BRITISH ARMY.—In the English Infantry the average number of sick is about 50 per 1000 men; in the English cavalry a little less; in the Royal artillery a little more; and the military train and depot battalions, at most 7,000 men, furnish about 1400 admissions per annum, on account of these two corps being chiefly composed of old and young soldiers. Striking an average in the British army the number of sick is nearly 55 per 1000 of strength; in the French army 45; in the Prussian 47; and in the Austrian 48. The average time in hospital is 17 to 20 or 21 days; in the French army it is 16 days; in the Prussian army it is 16 days; and in the Austrian army it is 17 days.

---

SCANZONI ON CHRONIC METRITIS.—This eminent physician has lately published a work on this subject, and dedicated to the Obstetrical Society of London. The author considers that one of the causes is *acute* metritis, the latter being due, among other causes, to excessive sexual intercourse. On this head Scanzoni finds great fault with the custom now prevalent in Germany, and imported from England, of traveling immediately after the wedding. He considers that Bennet, in England, and Becquerel, in France, are wrong in their views as to the pathology of chronic metritis, when these authors ascribe the develepement of different uterine affections to ulceration of the the cervix uteri. Scanzoni is by no means satisfied with the uterine sound; he warmly condemns its abuse, and considers the case very rare in which its use, in a diagnostic point of view, is indispensable.

---

PROPORTION OF BIRTHS TO POPULATION.—The proportion of births to population in various European countries is given in a blue-book of "Statistical Tables relating to Foreign Countries." In England and Wales the annual births are 1 in 28 persons; 1 in 30 in Belgium, Holland, and Norway; 1 in 32 in Sweden; 1 in 33 in Hanover, the Hans Towns, and Denmark; 1 in 34 in Greece; 1 in 38 in France; 1 in 26 in Wurtemburg; 1 in 25 in Russia; 1 in 24 in Austria, Saxony, and Prussia; and in Poland, 1 in 23.

Alleged Poisoning by Strychnine.—A crime similar to that committed by the notorious Palmer is the subject of judicial investigation in Paris. A physician insured the life of his wife for 500,000f. (£20,000), and shortly after the payment of the first premium the young woman died. The suddenness of the death, and the large amount for which the life was insured, created suspicion in the minds of the directors of the insurance company, and they determined to make the case known to the highest law authority. An investigation was commenced under the direction of the Imperial Attorney-General, in consequence of which the physician was arrested and committed to the prison of Mazas.

New Vaccination Act.—On the 1st proximo, an act of Parliament will come into force making it compulsory in Ireland to have all children born after the 1st of January vaccinated within six months, under a penalty of 10s.

Two New Cases of Syphilis Conveyed by Vaccination.—Besides the case of M. Devergie, lately mentioned, we have now one alluded to by M. Chassaignac before the Surgical Society of Paris; and another observed by M. Herard, and brought before the Medical Society of Hospitals. The parents, in both cases, have not suffered from syphilis, and the specific ulcers became apparent in the children at the spot where vaccination had been performed. The symptoms of syphilis were verified by the members of both the above-mentioned Societies.

The Queen and the Tobacco Question.—The use of tobacco within the precincts of Windsor Castle has been prohibited by express command of Her Majesty the Queen.

